# The dramatic development of X-ray photocrystallography over the past six decades

**DOI:** 10.1063/1.4975301

**Published:** 2017-02-01

**Authors:** Philip Coppens

**Affiliations:** Department of Chemistry, University at Buffalo, State University of New York, Buffalo, New York 14260-3000, USA

## Abstract

A short description of some of the paradigm-changing developments of the study of light-induced structural changes in molecular crystals is presented. The review is by no means comprehensive. The extensive literature on the subject should be consulted for further information.

## I. INTRODUCTION

**FIG. 1.**
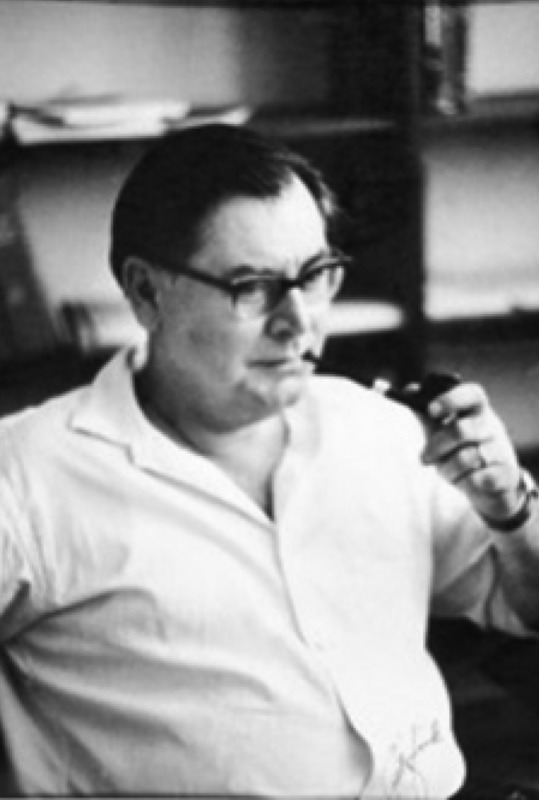
G. M. J. Scmidt in a typical pose.

The birth of photocrystallography deserves much credit to work in the fifties and sixties by Gerhard Schmidt and co-workers at the Weizmann Institute of Science in Israel. Gerhard Schmidt (Fig. 1) (Schmidt, 1971) who had just started to set up the first X-ray laboratory in Israel at the Weizmann Institute of Science maintained that X-ray crystallography was more than solving crystal structures and should be directed towards solving chemical problems. One of those involved the study of light-induced processes in crystals. Lasers, which were unknown at the time so the preferred light source was the Sun, “conveniently installed at 92 955 807 miles above the roof of the Weizmann Institute.” Schmidt described the preceding decades as the heroic era, in which hypotheses could not be based on the knowledge of crystal structures (Schmidt, 1971). Most of us would agree that the fifties and sixties were similar heroic areas, given, with hindsight, the primitive methods which had to be applied.

In the early crystallographic experiments, crystals would break down after a few percent conversion. Nevertheless is was possible to establish a firm relation between the crystal packing of cinnamic acids and their derivative, and the nature of the dimer produced by the photoreaction which would be the mirror-symmetric β-truxinic acid or the centro-symmetric α-truxillac acid leading to the term topochemical reactions, i.e., reactions governed by the topological relation between the reactants, a concept still valid with some exceptions due to defects in the crystals today.

The *topochemical postulate*, was first proposed by Kohlshutter in 1919 (Kohlschütter and Haenni, 1919) and systematically developed by Schmidt and co-workers with a new insight into molecular packing from X-ray diffraction. It states that chemical reactions in crystals proceed with a minimum amount of atomic motion (Cohen and Schmidt, 1964; Schmidt, 1964). Though exceptions have been found, it is clearly possible in many cases to predict the photo-induced reaction that will occur from the crystal structure of the reactants.

## II. EARLY STUDIES

During the following period, studies on 2 + 2 and other dimerizations continued with very much improved conventional sources such as mercury lamps, typically used with wavelength filters (Atkinson, 1970) (Cohen *et al.*, 1973; Cohen, 1975; Turowska-Tyrk, 2002; Turowska-Tyrk and Trzop, 2003). Noticeable are related studies of polymerization of diolefins and diacetylenes (Wegner; 1977; Hasegawa, 1983). The related multiple 2 + 2 isomerization of conjugated olefins leading among others to novel species, labeled ladderanes, was pioneered by MacGillavray and co-workers (Gao *et al.*, 2003; Friscic and Macgillivray, 2005).

## III. FOLLOWING DEVELOPMENTS

The development of photocrystallography was tremendously stimulated when lasers became readily available so that it became possible to select very well-defined wavelengths. The use of tunable lasers was strongly simulated in 1993, with the discovery by Enkelman and co-workers, that the [2 + 2] dimerization of cinnamic acid could be performed to a very high conversion percentage with preservation of the crystal lattice, by carefully irradiating in the tail of the absorption band (Enkelmann and Wegner, 1993). Further increase in expansion of the technique resulted from the application of two-photon processes, which allowed the avoidance of strong absorption bands at shorter wavelengths which may strongly restrict the penetration of the light beams in the crystal samples as illustrated in the photo-induced cleavage of coumarin photodimers (Kim *et al.*, 2003), by Harada, Nakajima, and Ogawa in a study of the photochromic ring closure of fungicides during which the crystal color changed from yellow to dark red (Harada *et al.*, 2008). And also in the controlled dimerization of α-cinnamic acid which also led to a study of the kinetics of crystal reaction (Benedict and Coppens, 2009).

## IV. PHOTOCHEMICAL REACTIONS IN SUPRAMOLECULAR SOLIDS

**FIG. 2.**
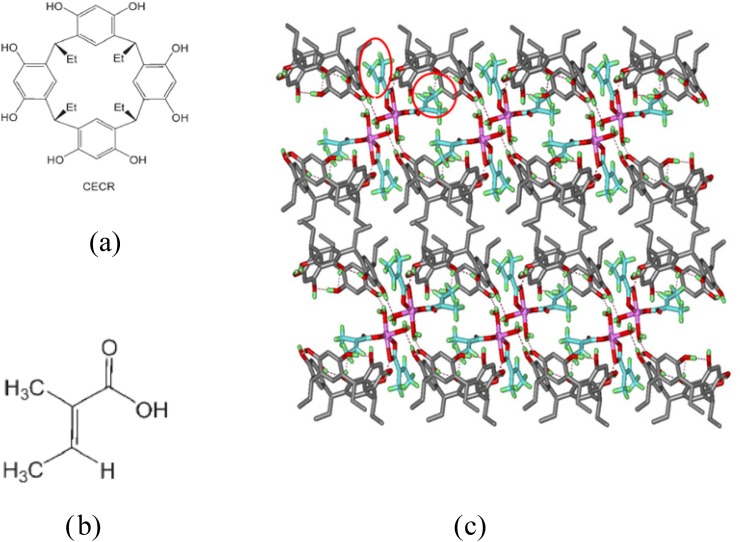
(a) C-ethyl[4]resorcinarene. (b) Tiglic acid. (c) 3D-supramolecular architecture of CECR-[Zn(TA)_2_ (H_2_O)_2_]·4H_2_O viewed along the b-axis direction (Zn atom is displayed as a sphere). Small and large cavities containing the Zn tiglic acid complex are circled. (Reproduced with permission from Zheng *et al.*, Chem. Eur. J. **14**, 706–713 (2008). Copyright 2008 John Wiley & Sons, Inc.)

As now widely recognized, supramolecular solids are an excellent medium for the study of photochemical reactions (Ramamurthy and Inoue, 2011) unless luminescence is quenched by energy transfer to linker molecules in the host framework (Zheng and Coppens, 2005a, 2005b). However, when the framework is composed of photo-inert host molecules, photosensitive species embedded in the cavities formed allow the study of slow reactions using steady-state techniques, in which the change is studied after successive exposures, often of hours. C-methyl[4]resorcinarenes and C-ethyl[4]resorcinarenes (Fig. 2) are particularly suitable framework formers for this purpose. They were used in our studies of the E → Z and Z → E (trans to cis and cis to trans) reactions of 3-chloroacrylic acid in C-ethylcalix[4]resorcinarene (CECR) (Zheng *et al.*, 2007) and a similar photoreaction of Zn tiglic acid CECR–[Zn(TA)_2_ (H_2_O)_2_]·4H_2_O.p inclusion compound in CECR (Zheng *et al.*, 2008). The latter is of particular interest, as the guest is contained in two cavities of different sizes, in which the reaction proceeds at different rates. Results performed at four different temperatures are shown in Table I.

**TABLE I.** Activation energy E_a_ and pre-exponential factor A for Z/E isomerization of the tiglic acid ligand in the small (S) and large (L) cavities of the inclusion complex CECR-[Zn(TA)_2_ (H_2_O)_2_]·4H_2_O.

	E → Z		Z → E	
	TA@S	TA@L	TA@S	TA@L
E_a_ [kJ mol^−1^]^a^	2.3(3)	1.8(1)	2.1(2)	1.9(1)
A [10^−3 ^s^−1^]^b^	1.18	1.16	4.05	1.36

^a^ Arrhenius expression: ln(k) = ln(A)−E_a_/RT.

^b^ Values of A only have a relative meaning, as they depend on the photon flux incident on the crystals and the absorbance.

The thermodynamic information on the crystal reactions summarized in Table I was derived from Arrhenius and Eyring plots. This is to our best knowledge, the first photocrystallographic study of kinetics in crystals (Fig. 2).

## V. SYNCHROTRON STUDIES OF FLEETING SPECIES WITH MICROSECOND TO PICOSECOND-LIFETIMES

With the advent of both high-brightness pulsed sources concentrating on a large photon flux in a small area and powerful pulsed lasers, previous limitations rapidly started to disappear, and it became possible to study the fleeting species with very short lifetimes. Third generation synchrotron sources initially the Synchrotrons the National Synchrotron Light Source at Brookhaven National Laboratory, Advanced Photon Source at Argonne National Laboratory, the European Synchrotron Radiation Facility in Grenoble France, and Spring8, Japan are now complemented by many new facilities.

Since the typical width of synchrotron sources is of the order of picoseconds, the study of excited molecular states and chemical reactions is controlled by this limit. However, this still opens a wide range of possibilities which include many luminescent inorganic and organic complexes, as well as biologically important reactions. The first time-resolved diffraction study of a fleeting species was performed at 17 K on (TEA)_3_H[Pt_2_(pop)_4_] with [pop = pyrophosphite, (H_2_P_2_O_5_)_2_, TEA = tetraethylammonium] (Kim *et al.*, 2002). The observed bond shortening of 0.28 (9) Å was within the experimental error equal to values determined spectroscopically from analyses of the IR spectra in crystals (0.21 Å) (Rice and Gray, 1983) and Raman spectra of an acetonitrile solution (0.225 Å) (Leung *et al.*, 1999). The value has since been confirmed by a value of 0.23 Å in a second diffraction experiment (Ozawa *et al.*, 2003), and more recently by scattering measurements on an aqueous solution (0.24 Å) (Christensen *et al.*, 2009) and a time-resolved EXAFS study (0.31 Å) (van der Veen *et al.*, 2008).

**FIG. 3.**
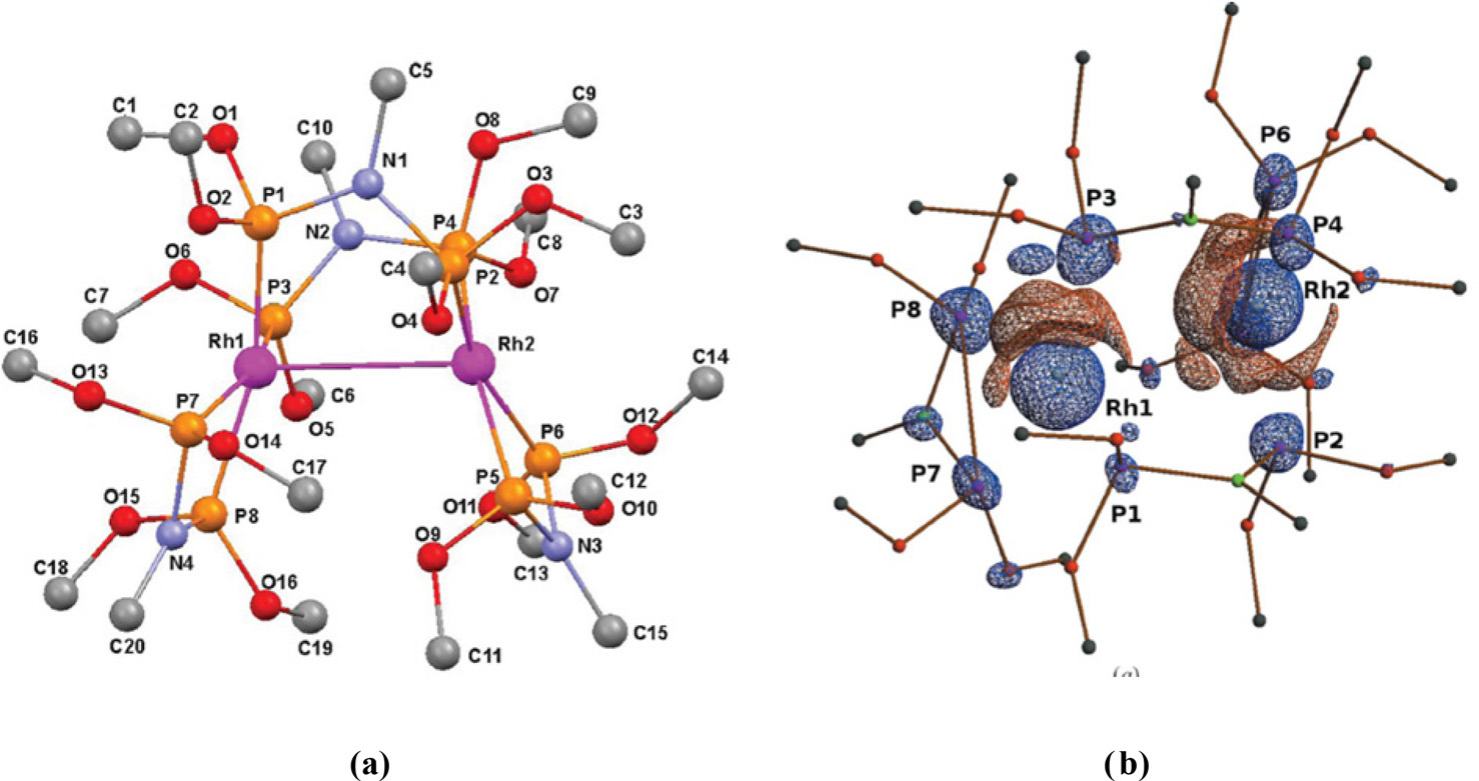
(a) Molecular diagram of Rh_2_(μ-PNP)_2_(PNP)_2_ (BPh_4_)_2_ [PNP = CH_3_N(P(OCH_3_)_2_)_2_, Ph = phenyl] and labeling of atoms. (b) Photodifference maps with isosurfaces (red positive, blue negative) of 0.25 e Å^−3^. (Reproduced with permission from Makal *et al.*, Acta Cryst. A **67**, 319–326 (2011). Copyright 2011 International Union of Crystallography.)

Additional examples of excited state studies of metalloorganic complexes are the 100 ps study of Rh_2_(μ-PNP)_2_(PNP)_2__BPh_4_ (Makal *et al.*, 2011) (Fig. 3), and the very large shortening of the Rh-Rh distance of 0.85 Å in [Rh_2_(1,8-diisocyano-p-menthane)_4_]^2+^ (Coppens *et al.*, 2004).

The initial studies at monochromatic sources were followed by the development of the “pink” Laue technique, first applied to the dynamics of biological systems (Ren and Moffat, 1995; Moffat, 2001), and more recently to the analysis of the structure of fleeting molecular triplet states (Makal *et al.*, 2011) (Fig. 3), and an Ag-Cu containing coinage metal complex (Jarzembska *et al.*, 2014).

The narrow band Laue technique makes much more efficient use of the available synchrotron photons and thus shortens the data collection time and increases accuracy if appropriate procedures are followed (Coppens and Fournier, 2015). The spectral assignment of the individual reflections can be bypassed in the time-resolved Laue work by use of the Ratio Method (Coppens *et al.*, 2009).

Other well-researched applications are the investigations of light-induced spin-cross-over processes as studied by, for example, Collet and Cailleau (Collet, 2010; Cailleau *et al.*, 2010) and Lecomte (Legrand *et al.*, 2006) and their co-workers.

## VI. THE NEW FRONTIER: SERIAL FEMTOSECOND STUDIES AT X-RAY FREE ELECTRON LASER SOURCES

The ultimate goal of photo-crystallographic studies of structural dynamics must include the initial states of chemical reactions which take place on femtosecond time scales. The development of tracking of chemical reactions by serial femtosecond pump-probe X-ray crystallography (SFX) in which the diffraction patterns are rapidly recorded at closely spaced time intervals, and sample decay is avoided by reducing the X-ray intensity, (Chapman *et al.*, 2011; Pande *et al.*, 2016; Spence, 2017) is of particular importance. It is now being applied at X-ray Free Electron Lasers (XFELs) such as LCLS at Stanford and SACLA at the Riken lab in Japan. It is clear from the results that the direction-controlling initial stages of chemical reactions occur below the 100 ps synchrotron limit. An example are the initial stages of the photoexcitation of photoactive yellow protein (PYP) which are triggered by the trans-cis isomerization of the coumarin chromophore (Tenboer *et al.*, 2014; Schmidt *et al.*, 2015; Spence, 2017). The amount of data collected in such studies is astronomical, for PYP 2.5 × 10^6^ snapshots were collected at 1.6 Å resolution in the PYP study.

## VII. FINAL REMARKS

**FIG. 4.**
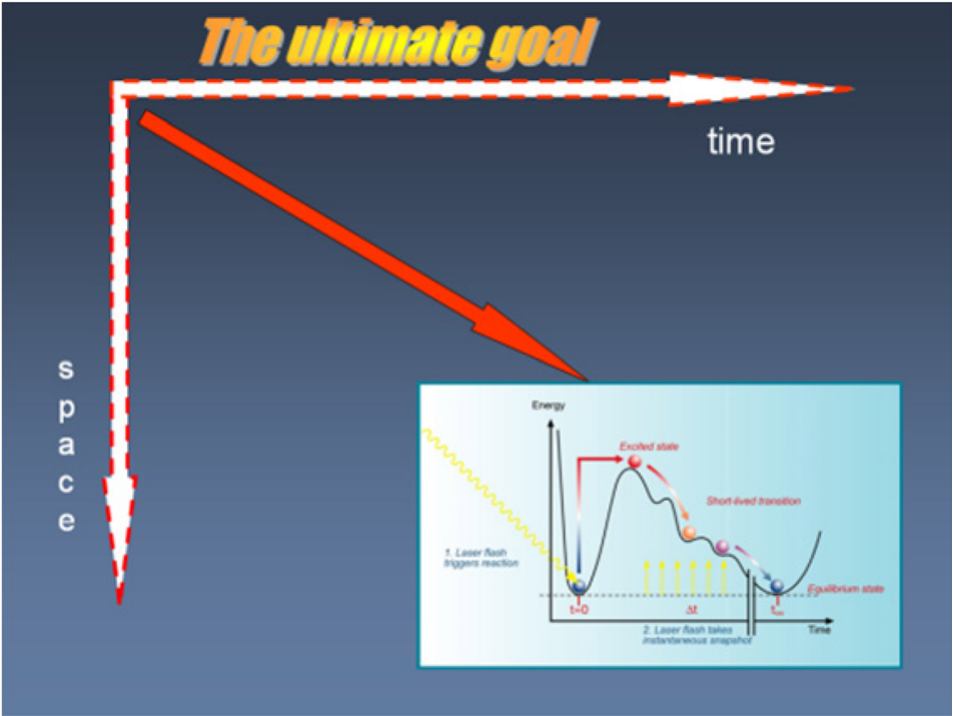
The ultimate goal!

The development of the field has been dramatic and impossible to predict in the early years. Photocrystallography is now a powerful technique spanning from time scales of hours to femtoseconds. Important dynamics take place in this whole span of ranges. I have emphasized that slower than femtosecond studies should not be ignored if a comprehensive picture of the structural dynamics of a system is to be achieved (Coppens, 2015). The ultimate goal is illustrated in Fig. 4.
